# Seven-Layer Model in Complex Networks Link Prediction: A Survey

**DOI:** 10.3390/s20226560

**Published:** 2020-11-17

**Authors:** Hui Wang, Zichun Le

**Affiliations:** 1 College of Computer Science and Technology, Zhejiang University of Technology, Hangzhou 310023, China; 1111712012@zjut.edu.cn; 2 College of Applied Science, Jiangxi university of Science and Technology, Ganzhou 341000, China; 3 College of Science, Zhejiang University of Technology, Hangzhou 310023, China

**Keywords:** complex networks, seven-layer model, link prediction, topological features

## Abstract

Link prediction is the most basic and essential problem in complex networks. This study analyzes the observed topological, time, attributive, label, weight, directional, and symbolic features and auxiliary information to find the lack of connection and predict the future possible connection. For discussion and analysis of the evolution of the network, the network model is of great significance. In the past two decades, link prediction has attracted extensive attention from experts in various fields, who have published numerous high-level papers, but few combine interdisciplinary characteristics. This survey analyzes and discusses the existing link prediction methods. The idea of stratification is introduced into the classification system of link prediction for the first time and proposes the design idea of a seven-layer model, namely the network, metadata, feature classification, selection input, processing, selection, and output layers. Among them, the processing layer divides link prediction methods into similarity-based, probabilistic, likelihood, supervised learning, semi-supervised learning, unsupervised learning, and reinforcement learning methods. The input features, evaluation metrics, complex analysis, experimental comparisons, relative merits, common dataset and open-source implementations for each link prediction method are then discussed in detail. Through analysis and comparison, we found that the link prediction method based on graph structure features has better prediction performance. Finally, the future development direction of link prediction in complex networks is discussed.

## 1. Introduction

Networks comprise a series of nodes and edges and can describe many systems in the real world. Nodes usually represent entities in the system, and links represent relationships or activities between entities. For example, if a node represents a person and an edge represents an interaction between two nodes, it can form a social network reflecting interpersonal relationships. If the nodes represent cities and the edges represent railways between cities, we can build a railroad network, reflecting the status of traffic routes. Examples of this can be seen everywhere. Therefore, the research of network science has attracted the attention of an increasing number of experts. As an important research direction in network science, link prediction can use existing network information to predict lost and new links that might be deleted or added in the future. It can be used in a recommendation system to help people find new partners or potential friends and provide products of interest in online shopping. It can also be used to infer the complete network structure and better understand the evolution of the network.

**Figure 1:**
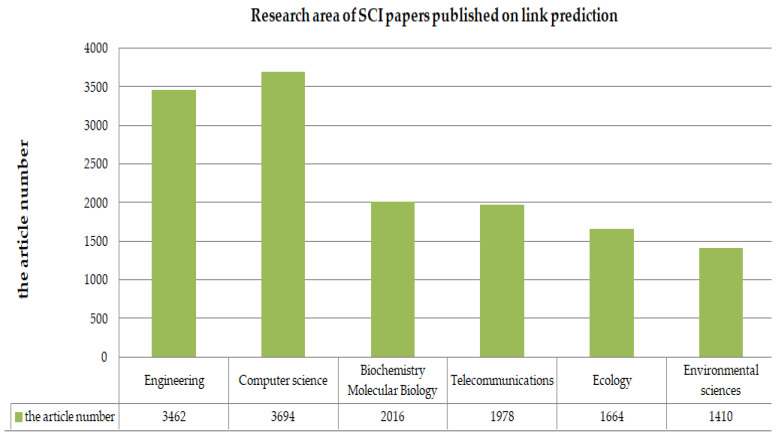
Research area of SCI papers published on link prediction.

**Figure 2:**
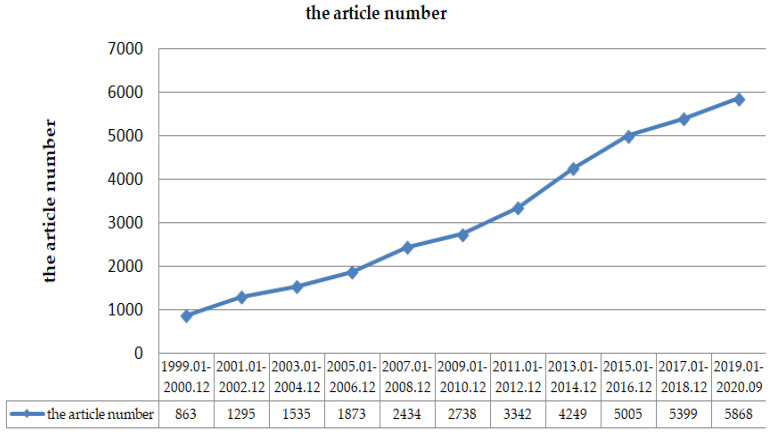
The number of papers published on link prediction.

**Figure 3:**
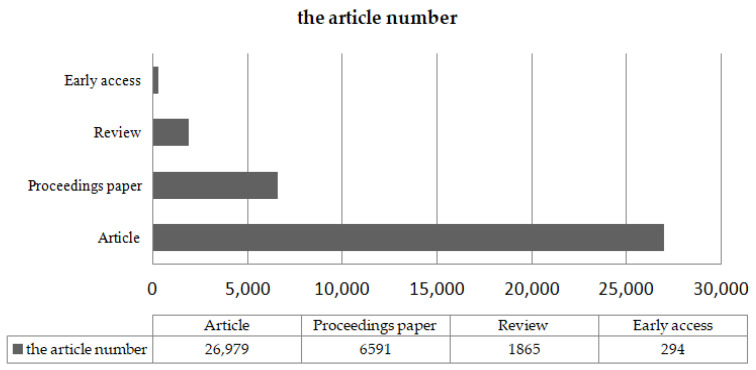
Type of SCI papers published on link prediction.

**Figure 4:**
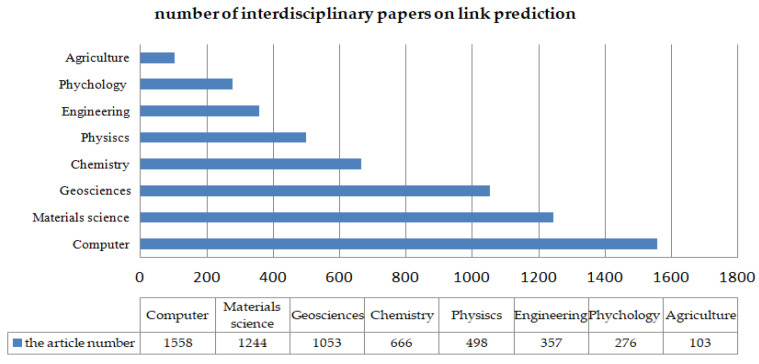
Number of interdisciplinary papers on link prediction.

Between 1999 and 2020, engineering scientists, computer scientists, biochemist, telecommunications, biologists, geneticists, and environment scientists have made many attempts to solve the link prediction problem (Figure 1). Figure 2 shows the total number of published papers searched on the web of science with the topic of link prediction between 1999 and 2020. An increasing number of articles focus on link prediction, with thousands written every year on the subject. Although from 1999 to 2020, the number of papers published on link prediction keeps increasing every year, with 37,279 papers, few are interdisciplinary papers, especially review papers. Figure 3 shows that it was less than 5%. As shown in Figure 4, only 15% of multidisciplinary link prediction papers have been published from 1999 to 2020. Among them, 15,558 were from computer multidisciplinary, 1244 were from materials science multidisciplinary, 1053 were from geosciences multidisciplinary, and 666 were from physics multidisciplinary. The above data analysis shows that there are few review papers on interdisciplinary link prediction.

According to cited frequency, from 1999 to 2020, some excellent research reviews of link prediction were published. Liben-Nowell et al. [1] published a seminal review paper in 2007, receiving around 5 thousand citations at the mid of 2020 on baidu academic. This study proposes a link prediction method based on the proximity measure of nodes in co-authorship networks. Lü et al. [2] also published a review paper, obtaining around 2000 citations. The paper reviews the research progress of link prediction methods in recent years, including random walk approaches and maximum likelihood approaches. In 2019, Shang et al. [3] divided link prediction methods into traditional link prediction methods and link prediction methods based on tree networks. Although these articles have received some attention, they only use physical methods to analyze the link prediction problem, not informatics methods. That is to say, they are analyzed from the perspective of feature extraction rather than from the perspective of feature learning.

Hasan et al. [4] published a review paper in 2011, receiving around 400 citations at the mid of 2020 on baidu academic and considered three types of models for social networks, namely no-bayesian models, probabilistic approaches, and linear algebraic approach. Wang and Wang [5] published a paper in 2015, obtaining around 200 citations, proposing link prediction approaches based on learning and topology. This study analyzed in detail link prediction from the perspective of physics and informatics. Although the above paper is valuable and meaningful for link prediction research, they lack the research on the latest link prediction technology, especially the most popular deep learning link prediction technology based on graph features. In 2020, Daud et al. [6] published a more comprehensive review paper, involving the classification and application of link prediction, but only focuses on social networks. Furthermore, most research results are for static networks, and the review of link prediction for dynamic and heterogeneous networks is few. However, the real network was complicated, not only static networks. Currently, we need a comprehensive review paper, focusing on the latest link prediction techniques including link prediction methods to solve different networks.

To compensate for the deficiencies of existing review papers, this study provides a systematic overview of link prediction from static to dynamic networks, from homogeneous to heterogeneous networks, and from unsigned to signed networks. First, it formally defines link prediction. The hierarchical design idea is introduced into the link prediction classification system from the perspective of informatics and physics. The hierarchical model consists of seven layers, namely the network, metadata, feature classification, selection input, processing, selection, and output layers. Then, link prediction techniques commonly used in various fields are discussed from two parts and seven aspects. The two aspects include feature extraction and feature learning methods. The seven aspects refer to similarity-based, probabilistic, likelihood, unsupervised learning, semi-supervised learning, supervised learning, and reinforcement learning methods. These methods include many classic and up-to-date link prediction techniques. Algorithm complexity, input feature, relative merits, common dataset and experimental comparison are also described. Finally, based on the summary of existing research results, the future research direction is proposed.

The organizational structure of this paper is as follows. In Section 2, we define link prediction. Section 3 proposes the seven-layer model, explaining the specific functions of each layer. The classical and up-to-date link prediction approaches used in various fields are introduced and classified. Section 4 discusses the input feature of common link prediction methods and analyzes the complexity of the algorithm. Section 5 introduces evaluating metrics, and in Section 6, we summarize the open-source implementation for the common link prediction methods. In Section 7, we compare the experiments and advantages of the link prediction algorithms involved in this article. At the same times, we provide common dataset. Finally, we summarize the future development direction of link prediction.

## 2. Problem Formulation

The formal definition of the link prediction problem is discussed below. In the network G(V,E,T,W), V represents the set of nodes, E shows the set of edge and weight set W at time T. v_i_ (i = 1,2, …, n) is the union of different node sets, E_j_ (j = 1,2, …, m) is the union of different edges sets, T_k_ (k = 1,2, …, u) is the union of different time sets, and W_z_ (z = 1,2, …, v) is the union of different weight sets. Link prediction must output a list of edges and weight not existing in G(t_0_, t_i_), but it was predicted to show up in the network G(t_j_) for t_0_ < t_i_ < t_j_. If i > 1 or j > 1, it means that the nodes and edges in the network are of different types, which we call a heterogeneous network. Otherwise, it is a homogeneous network.

## 3. Link Prediction Seven-Layer Model

**Figure 5:**
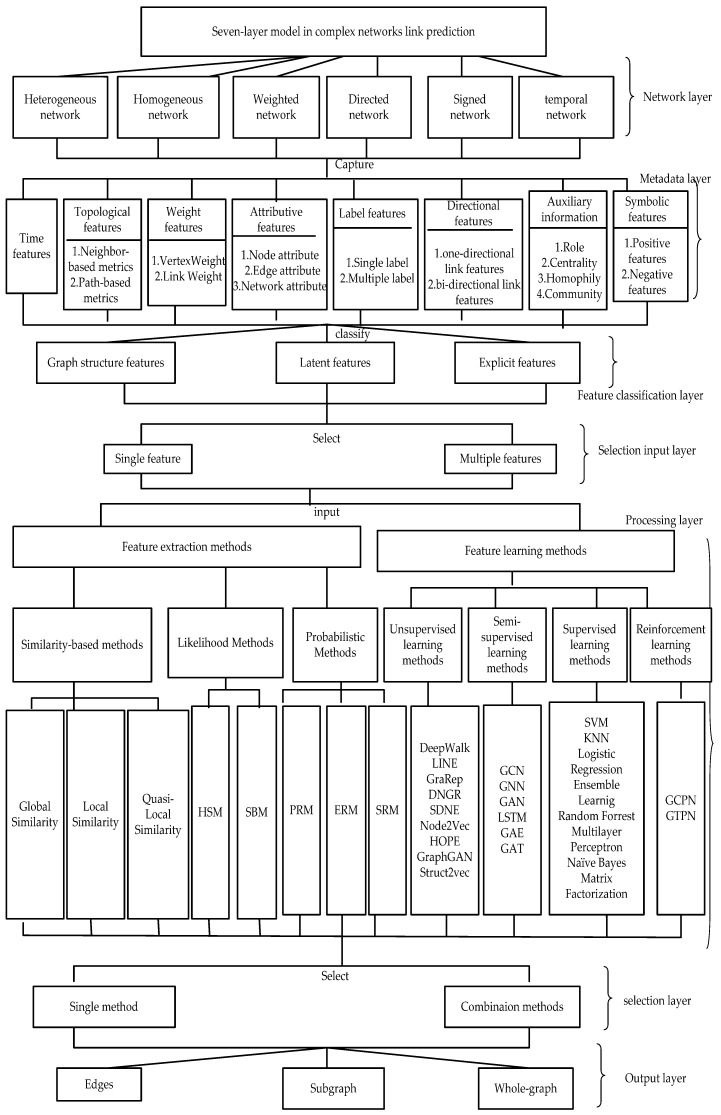
Seven-layer model in complex networks link prediction.

**Figure 6:**
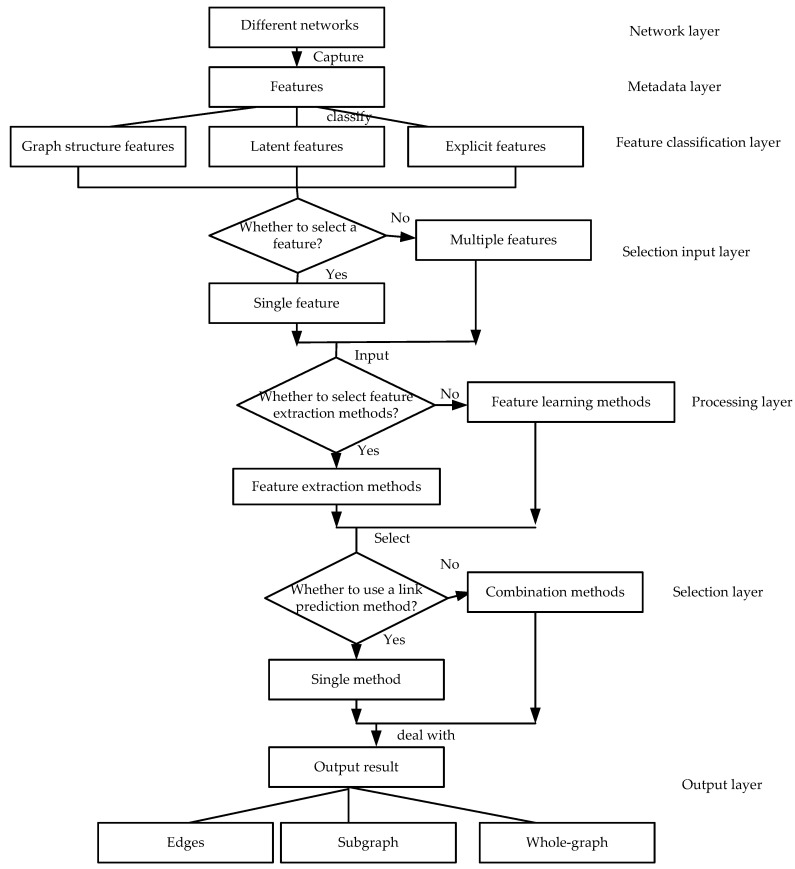
The data flow graph of the seven-layer model.

This study classifies these commonly used, classical and latest link prediction methods in complex networks by proposing a new link prediction hierarchical classification reference model. Figure 5 shows our proposed link prediction hierarchical classification reference model, which is divided into seven layers from top to bottom. The data flow graph of the seven-layer model (the relationship between each layer) is shown in Figure 6. Each layer and its functions are described in detail below.

### 3.1. Network Layer

The network layer is located at the bottom of the link prediction seven-layer model in complex networks, including five networks involved in the research of link prediction, namely the heterogeneous, homogeneous, weighted, directed, signed, and temporal networks.

Currently, most review papers on link prediction focus on static and homogeneous networks, which means the types of nodes and edges being single. However, the real network is complex, consisting of different types of nodes or links, which might differ in weight, direction, symbolically, contents, and attributes. Therefore, the network layer design should be as comprehensive as possible, including all networks participating in link prediction research so far.

### 3.2. Metadata Layer

The metadata layer sits at the second level of the seven-tier model in complex networks. This layer has many basic features, including topological, time, attributive, label, weight, directional, and symbolic features, and auxiliary information as input metadata to predict the probability of two nodes links.

#### 3.2.1. Time Features

The actual network is dynamic and traditional network analysis tools cannot effectively mine the deep information of the network. It is critical to use time features as input metadata for link prediction, especially of dynamic networks. The time features can infer the hidden information of the network according to historical information.

Munasinghe [7] introduced the time score (TS) as a new input feature, which analyzes the influence of time factor on link prediction strength. The author considered the temporal awareness of link prediction, using the TS as a new input feature to predict possible links in the future. The prediction performance is significantly improved. Huang et al. [8] introduced the time-series feature, considered capturing the time evolution features of link occurrences, and predicted the link occurrence probability at a specific moment. The time-series dynamic link prediction method is superior to the static graph link prediction method. Ricardo et al. [9] proposed that introducing time as an input feature could significantly improve performance for link prediction. By calculating the similarity scores of non-connected nodes at different time points, the author established time-series features for each pair of disconnected nodes, deployed the prediction model on these time-series features, and took the prediction result as the final scores of the future link probability. Xu et al. [10] proposed the concept of the active factor using time features, and extended the link prediction framework based on similarity.

#### 3.2.2. Topological Features

Most of the input metadata are based on topological features because it is simpler and much more convenient to obtain the topology of the node than attribute information. The topology-based link prediction method has universal applicability in various networks.

Liben-Nowell and Kleinberg [1] studied structural features of graphs and proposed many topology-based features in succession [11], which are divided into neighborhood-based and path-based features. The links are both direct and indirect. Wang et al. [12] believed that neighborhood-based features perform best. The node similarity methods only needed information about the nearest neighbors to achieve a high score and low computational complexity, especially for networks with low clustering coefficients, which are efficient and simple. In 2017, Shang et al. [13] found that the direct link method performs better than the indirect link method for a range of time-varying networks and verified that topology-based features are more significant. Neighborhood-based features are also especially critical.

#### 3.2.3. Weight Features

Actual networks have the features of interactive weights. The existing link prediction approaches are mainly used for simple unweighted static networks without considering the influence of weights, so they cannot be adapted to the case of complex networks. We considered weight features as input data for link prediction being necessary. By predicting the link and weight of the network, we can reconstruct the network model with good recovery of missing links. The weight feature includes vertex and link weight. Vertex weight is the degree value of the target vertex minus the degree value of the inactive vertex connected to the target node and error link. Link weight is defined as the degree values of two endpoints minus a low-weight link. Vertex weight tends to be the attribute features of the network, whereas link weights tend to be the global features of the network. Danh et al. [14] used local paths with weight features to solve link prediction problems, which had better predictions.

#### 3.2.4. Attributive Features

Most link prediction methods used in the computer field are Markov chains and machine learning. These methods take attribute features as input metadata and obtain high prediction accuracy. If the attributes of the node pairs are similar, they will be more likely to connect in the future, mainly including node, edge, and network attribute features. Shi et al. [15] proposed two new measures of affective similarity and designed an algorithm for link prediction combining node topological features and affective attribute features. The results suggest that if two people share the same emotions, they are more likely to connect in the future. Zhang et al. [16] proposed a new random walk link prediction method combining topological and attribute features. Gong et al. [17] used the random walk method with a restart to combine topological and attribute features to conduct link prediction of social networks, which improved link prediction performance to a certain extent.

#### 3.2.5. Label Features

In complex networks, label features can also be used to predict the basic features of links, including single and multiple labels. When an object belongs to one of several candidate classes, it is called a single label, and when an object belongs to multiple categories in multiple candidate classes, it is called a multiple label. Zhao et al. [18] proposed a new multi-label relational neighbor classifier for link prediction based on content features, which calculates the weight of links according to the similarity of social features between nodes and predicts the missing links. The multi-label relationship classifier is refined by discovering potential relationships between nodes.

#### 3.2.6. Directional Features

Most original link prediction methods use the network topology to predict whether there will be a link relationship between nodes. The influence of direction features on link formation is seldom considered. However, real-world networks have directions, and ignoring the direction of the link means ignoring critical information. Directional features include one-directional and bi-directional link features. Compared with the node pair with the bi-directional link, it can provide more information about network structures and has better predictive performance than the one-directional link. Shang et al. [19] proposed that links in different directions play different predictive roles. The directional features are significant in link prediction and can provide more potentially useful information. In random networks, the ratio of one-directional and bi-directional links will determine the accuracy of link prediction.

#### 3.2.7. Symbolic Features

With the development of signed networks, Thi et al. [20] proposed the significant influence of symbolic features on link prediction, in which symbolic features reflect positive and negative links, such as likes and dislikes. In this framework, the positive/negative ratio features are proposed for edge symbolic prediction.

#### 3.2.8. Auxiliary Information

**Figure 7:**
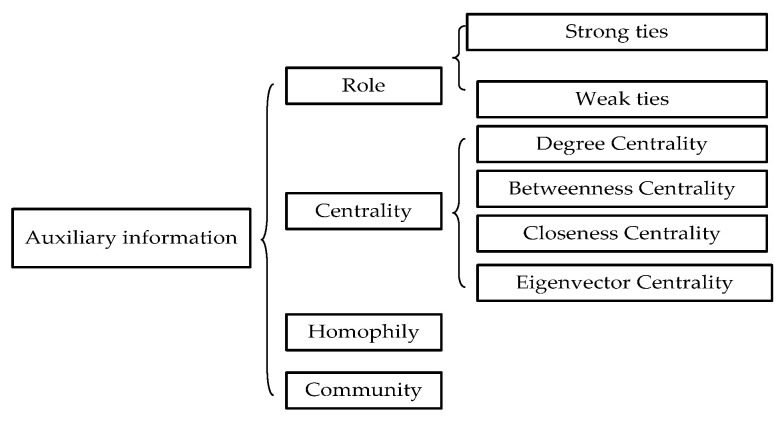
Auxiliary information graph.

Recently, an increasing number of studies use auxiliary information, such as role, centrality, homophily, community, and structural balance, to evaluate and analyze the complex network. Early link prediction methods only used topology, time, attribute, label, weight, and directional features. However, using auxiliary information as input metadata for link prediction could mine potentially useful information and improve prediction performance (Figure 7).

1. Role
  As auxiliary information in link prediction, role mainly includes strong and weak ties. Strong ties are stable and deep social relations, whereas weak ties are flexible and extensive social relations compared with strong connections. In social networks, approximately 20% are strong and 80% are weak relational connections. Weak ties are more crucial than strong ties. Liu et al. [21] claimed that weak ties significantly influence link prediction, and the performance of link prediction can be improved using weak ties.
2. Centrality
  In many real networks, centrality theory also significantly influences the performance of link prediction. Nodes in a network prefer to link to both similar and central nodes. Li et al. [22] used a maximum entropy random walk for link prediction, and the method used the node centrality theory, which had better performance than the link prediction method without centrality theory. Ma et al. [23] proposed a centrality information model, improving the performance of link prediction using node importance theory. It included several centralities, such as degree, closeness, betweenness, and eigenvector centrality.
3. Homophily
  Wang et al. [24] found the internal relationship between links and attributes in the network by using the homogeneity theory, combining link prediction and attribute inference with the community structure, and proposed a community-based link and attribute inference approach. This method can both predict attributes and links and improve the accuracy of link prediction and attribute inference by the iterative method. In social networks, Yang et al. [25] proposed a model that uses the homophily theory to connect users with the services in which they are interested and connect different users with common interests to effectively spread friendship and interests. Weng et al. [26] describe the user with a set of parameters associated with different link creation strategies and use maximum likelihood estimates to confirm that triadic closure does have a strong effect on link formation.
4. Community
  Taking the community structure information of the network as the input metadata can more easily discover some hidden laws in the network and predict the behavior of the network, help us further analyze the network topology, and better understand and explain the network functions. Weng et al. [27] propose a practical method that converts data about community structure into predictive knowledge of information that can be widely disseminated. Valverde-Rebaza and Lopes [28] combine topological structure and community information, have high efficiency, and improve the link prediction performance of directed and asymmetric large-scale social networks.

### 3.3. Feature Classification Layer

The feature classification layer is in the third layer of the seven-layer model in complex networks. Its function is to classify the features provided by the previous metadata layer and divide them into three categories, namely graph structure, latent, and explicit features. These three features are largely orthogonal to each other. Many papers have considered using them together for link prediction to improve the performance of single-feature-based methods.

1. Graph structure features
  Graph structure features are located in the observation nodes and edge structures of the network, which can be directly observed and calculated. Link prediction heuristics belong to graph structure features, such as Common Neighbors, Jaccard, preferential attachment, Adamic-Adar, resource allocation, Katz, PageRank, and SimRank. In addition to link prediction heuristics, degree centrality, closeness centrality, betweenness centrality, and eigenvector centrality belong to graph structure features, which are inductive, meaning that these features are not associated with a specific node or network. Cukierski et al. [29] used 94 distinct graph features as input metadata for classification with RF, at the same times, proposed several variants of similarity method for link prediction. The research shows that the combination of features can achieve a better prediction effect.
2. Latent features
  A latent feature is a potential attribute or representation of a node, usually obtained by decomposing a specific matrix derived from a network. They are powerful in linking predictions. Assume that each entity is associated with an unobserved eigenvector, the probability of the link is then calculated by the interaction between these potential features. They reveal structural relationships between entities, automatically learn potential features, make accurate predictions, and perform at their best. Latent features focus more on global properties and long-term effects, fail to capture structural similarities between nodes, and are less interpretable than graph structure features.
3. Explicit features
  Explicit features are usually given by continuous or discrete node attribute vectors. In principle, any side information about the network other than its structure can be seen as explicit features. For instance, in social networks, a user’s profile information is also an explicit feature. However, their friendship information belongs to graph structure features.

### 3.4. Selection Input Layer

The selection input layer is located in the fourth layer of the seven-layer model in complex networks. This layer performs link prediction by selecting single or multiple features of the upper layer as input elements. It includes single feature and multiple features.

1. Single feature
  Early link prediction approaches used a single feature in the classification layer for link prediction, that is, graph structure features, latent features, and explicit features only use one item as an input feature item.
2. Multiple features
  The graph structure, latent, and explicit features are largely orthogonal to each other. We can try using them together for link prediction to improve the performance of single-feature-based methods, that is, using a combination of graph structure and latent features or a combination of latent and explicit features. Koren et al. [30] established a more accurate composite model. The user’s explicit and implicit features are used to further improve the precision.

### 3.5. Processing Layer

The processing layer is in the fifth layer of the link prediction model. In this layer, the commonly used link prediction methods are divided into feature extraction and feature learning methods. When using the feature extraction method, the next step is to assign scores to node pairs of the target link type. If the score of a node pair exceeds a given threshold, the pair is likely to form a link in the future and vice versa. In feature learning methods, the link prediction problem is a binary classification problem. Two aspects, feature extraction and feature learning, are used to classify the link prediction methods in various fields.

#### 3.5.1. Feature Extraction Methods

Feature extraction methods are common link prediction methods in physics, including similarity-based, probabilistic, and likelihood methods [5].

1. Similarity-based methods

**Figure 8:**
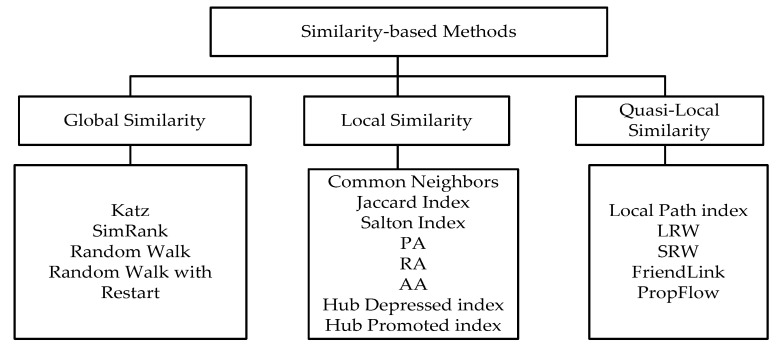
Similarity-based methods.

Similarity-based methods are the most used link prediction methods, which are easy to implement. The core idea is that if two nodes are more similar in structure, etc., the possibility of connecting the edges between them is greater. We set the node’s score for X and Y to be Sxy, and defined it as the similarity between node X and Y. Everything no-connected links is to calculate rank in complex networks according to their scores, where higher scores and high ranking nodes mean that x and y are more likely to be linked in the future. In this part, we divided 17 types of similarity-based indicators into three categories, among which Katz, SimRank, random Walk, and Random walk with restart are global in similarity. Common neighbors, Jaccard index, hub depressed index, Salton index, preferential attachment (PA), resource allocation (RA), and hub promoted index are local in similarity. Local path index, local random walk (LRW), superposed random walk (SRW), FriendLink, and PropFlow are quasi-local in similarity, which does not require global topology information, but uses more information than local similarity (Figure 8).

(1) Global Similarity

Different from the method based on local similarity, global similarity indices use the topological information of the entire network to sort the node pairs. Although considering the topology of the entire network, the link prediction analysis is more flexible, but it also increases the time complexity. Their time complexity and high degree of parallelism make them unsuitable for large networks, especially in distributed environments, the effect is poor.

- Katz Index (KI)

The Katz metric calculates the set of all paths between two nodes and incrementally processes the paths according to their length [31]. That is to say, the short path is given a larger weight, while the long path is given a smaller weight. This index is defined as

(1) $$S_{\left(x,y\right)}^{\mathrm{Katz}\;}=\sum_{i=1}^{\infty }\beta^{i}\left|{\mathrm{paths}}_{\mathrm{xy}}^{\langle i\rangle }\right|=\;\sum_{i=1}^{\infty }\beta^{i}{\left(A^{i}\right)}_{x,y}\;=\;ßA_{x,y}\;+{\;\beta }^{2}{\left(A^{2}\right)}_{x,y}\;+{\;\beta }^{3}{\left(A^{3}\right)}_{x,y}\;+\;\ldots ,$$

where ${\mathrm{paths}}_{\mathrm{xy}}^{\langle i\rangle }$ is a collection of all paths with length i from x and y. The free parameter ß is a damping factor, which controlled the path weights (ß > 0). If ß is very mall, it will cause the Katz metric to be close to the Common Neighbors (CN) metric, since paths of long length contribute very little to final similarities.

- SimRank (SR)

The definition of SimRank is self-consistent [32], holding that if two nodes are connected to similar nodes, they are similar. This method is suitable for directed or mixed networks.

(2) $$S(x,y)\;=\;ß\frac{\sum_{i\epsilon \tau_{x}}\sum_{j\in \tau_{y}}s\left(i,j\right)}{\left|\tau_{x}\right|\left|\tau_{y}\right|},$$

where s(z,z) = 1 and 0 < ß < 1 is the decay factor. The SR is a random walk process, meaning that S(x,y) is used to describe when two particles starting from nodes X and Y, respectively, meet.

- Random Walk (RW)

Given a graph and starting node, we randomly select any neighbor of the starting node to move it, and then repeat the process for each arriving node [22], namely a random walk on the graph. If we define $\vec{p^{x}}$ as the probability vector to any node that goes on a random walk from node x, the probability of reaching each vertex can be iteratively approximated by

(3) $$\vec{p^{x}}(t)\;=\;M^{T}\vec{p^{x}}(t\;-\;1).$$

- Random Walk with Restart (RWR)

RWR directly applies PageRank’s algorithmic ideas to this method [17], and its assumption is that the random walk particle returns to the initial position with a certain probability every step. This model is known as a RWR. We have

(4) $$\vec{q_{x}}\;=\;{\mathrm{aP}}^{T}\vec{q_{x}}\;+\;(1\;-\;a)\vec{e_{x}},$$

where P is the transition probability matrix when P_xy_ = 1/k_x_, if x and y are connected, and P_xy_ = 0. Otherwise, the solution is straightforward as

(5) $$\vec{q_{x}}\;=\;{\left(1\;-\;a)(1\;-\;{\mathrm{aP}}^{T}\right)}^{-1}\vec{e_{x}}.$$

Therefore, the RWR index is thus defined as

(6) $$S_{\mathrm{xy}}^{\mathrm{RWR}}\;=\;q_{\mathrm{xy}}\;+\;q{\mathrm{yx}}_{,}$$

where $\vec{{\;e}_{x}}$ represents the initial state. q_xy_ is the probability that a particle starting from node x ends up at node y. Tong et al. [33] proposed a quick solution for computing the RWR. These methods use the community-like structure and linear correlations in the real graph adjacency matrix, using the Sherman-Morrison Lemma. This approach can accurately balance the off-line processing cost and quality of online responses.

(2) Local Similarity

Because only local topology information is used instead of the entire network, the prediction accuracy of similarity approaches based on local information is lower than global approaches. However, the lower computational complexity makes local approaches more applicable in many large-scale networks. They are faster than methods based on global similarity, especially on dynamic networks. Local approaches refer to those that can only be calculated through local information of nodes, which includes CN, JC, SI, PA, adamic-adar (AA), RA, HDI, and hub promoted index (HPI).

- Common Neighbors (CN)

For each pair of nodes, if they have a high CN number, they are more likely to have a link. These algorithms have been applied to many networks. CN is a method with low time complexity and easy implementation.

(7) $$\mathrm{CN}(x,y)\;=\;\left|\tau \left(x\right)\cap^{\;}\tau \left(y\right)\right|,$$

where τ(x) is the set of neighbors of node x. Newman [34] applied the CN method to the co-authorship network and found that the more CNs there are, the more likely it is for two authors to cooperate. Kossinets [35] applied the CN method to a large-scale social network, where two people have many friends in common and the possibility of becoming friends in the future is significant.

- Jaccard Index (JC)

More than 100 years ago, Paul Jaccard proposed the Jaccard method to compare the similarity and diversity of objects [36], showing the percentage of the two nodes’ shared neighbors in the entire neighborhood collection. The formula is defined as

(8) $$s_{\mathrm{xy}}^{\mathrm{Jaccard}}\;=\;\frac{\left|\tau \left(x\right)\cap^{\;}\tau \left(y\right)\right|}{\;\left|\tau \left(x\right)\cup^{\;}\tau \left(y\right)\right|}.$$

Bhawsar et al. [37] proposed the fuzzy soft-set-based link prediction model, which used various features of social networks. The Jaccard coefficient is combined with the fuzzy soft-set to predict the exact link, which is superior to the existing link prediction methods, such as CN and Sorenson.

- Salton Index (SI)

The core idea of this link prediction method is to measure whether two nodes are similar by common cosine similarity between nodes x and y [38].

(9) $$s_{\mathrm{xy}}^{\mathrm{Salton}}\;=\;\frac{\left|\tau \left(x\right)\cap^{\;}\tau \left(y\right)\right|}{\sqrt{k_{x}\times k_{y}}}$$

- Preferential Attachment Index (PA)

According to the principle of priority connection mechanism, the higher the degree, the more likely it is to generate links, and the lower the degree, the less likely it is to generate links [39], leading to the notion of the rich get richer. K_x_ refers to the degree value of node x.

(10) $$s_{\mathrm{xy}}^{\mathrm{PA}\;}=\;K_{x}\;\times \;k_{y}$$

- Adamic-Adar Index (AA)

In 2003, Adamic and Adar proposed a new similarity measure to calculate the similarity between two web pages [40]. It calculates the CN by assigning more weight to the neighbor with fewer connections.

(11) $$s_{\mathrm{xy}}^{\mathrm{AA}}\;=\;\sum_{z\in \tau \left(x\right)\cap^{\;}\tau \left(y\right)}\frac{1}{{\mathrm{logk}}_{z}}$$

- Resource Allocation Index (RA)

RA is a new measurement standard for network resource allocation [41], with a similar metric to AA. The biggest difference lies in the different ways of assigning weight to the CN node, which decreases at different rates, i.e., one is 1/k, and one is 1/lgk. If the degree is small, RA and AA have little difference, but when the average degree is large, the difference is large.

(12) $$s_{\mathrm{xy}}^{\mathrm{RA}\;}=\;\sum_{Z\in \tau \left(x\right)\cap^{\;}\tau \left(y\right)}\frac{1}{K_{Z}}$$

- Hub Depressed Index (HDI)

HDI is a link prediction method proposed by Zhou [42], which is like HPI. This similarity function can be defined as

(13) $$s_{\mathrm{xy}}^{\mathrm{HDI}}\;=\;\frac{\left|\tau \left(x\right)\cap^{\;}\tau \left(y\right)\right|}{\max\left\{k_{x},k_{y}\right\}}$$

- Hub Promoted index (HPI)

HPI [43] is used to quantitatively describe the topological similarity degree of each pair of reflectors in the metabolic network. This measure is defined as

(14) $$s_{\mathrm{xy}}^{\mathrm{HDI}}\;=\;\;\frac{\left|\tau \left(x\right)\cap \tau \left(y\right)\right|}{\min\left\{k_{x},k_{y}\right\}}$$

(3) Quasi-local Similarity

Local similarity has low time complexity but low precision. However, global similarity is the opposite. Therefore, an increasing number of researchers started to explore how to compromise the accuracy and time complexity of approaches to design more efficient link prediction methods. Quasi-local similarity emerged, striking a balance between local and global similarity. It includes local path (LP), local random walk (LRW), superposed random walk (SRW), FriendLink (FL), and PropFlow predictor (PFP).

- Local Path Index (LP)

The LP method was developed based on KI [44]. The biggest difference between LP and KI is that it considers local paths of length 2 and 3. It is defined as

S^LP^ = A^2^ + εA^3^. (15)

There is a free parameter ε. When ε = 0, LP = CN. Here A^2^ and A^3^ represent the adjacency degree of nodes with 2 and 3 lengths, respectively.

- Local Random Walk (LRW)

The proposal of LRW is based on the random walk of finite steps [45], which was suitable for large and sparse networks. q is the initial configuration function, the similarity based on the t-step random walk can be defined as

(16) $$s_{\mathrm{xy}}^{\mathrm{LRW}}(t)\;=\;q_{x}\pi_{\mathrm{xy}}\left(t\right)\;+\;q_{y}\pi_{\mathrm{yx}}\left(t\right).$$

- Superposed random walk (SRW)

SRW [45] summarizes the T-step and previous results based on LRW. The goal is to connect as many nodes adjacent to the target node as possible to the target node. It is defined as follows:

(17) $$s_{\mathrm{xy}}^{\mathrm{SRW}}(t)\;=\;\sum_{\tau \;=\;1}^{t}s_{\mathrm{xy}}^{\mathrm{LRW}}(\tau )\;=\;\sum_{\tau \;=\;1}^{t}\left[q_{x}\pi_{\mathrm{xy}}\left(\tau \right)+q_{y}\pi_{\mathrm{yx}}\left(\tau \right)\right].$$

- FriendLink (FL)

FL [46] is a new measurement method, measuring the path number of interested nodes, which is similar to LP. The difference is that this method uses normalization and other path length penalty mechanisms. The prediction effect is better and more accurate, which is defined as

(18) $$S_{\mathrm{xy}}^{\mathrm{FL}}\;=\;\sum_{i\;=\;1}^{l}\frac{1}{i-1}\cdot \frac{\left|{\mathrm{paths}}_{x,y}^{i}\right|}{\prod_{j\;=\;2}^{i}\left(n-j\right)}.$$

In the above formula, n shows how many nodes are in the network. l represents the path length between node x and y, and the set of all paths of length i from x to y is ${\mathrm{paths}}_{x,y}^{i}$.

- PropFlow Predictor (PFP) Index

PropFlow [47] is inspired by PageRank and calculates information between nodes through a LP to measure whether a link will occur. The size of the PropFlow value determines the likelihood of future links, and the larger the value, the easier it is to generate links. PropFlow is now widely used in directed, undirected, weighted, unweighted, sparse, or dense networks.

(19) $$s_{\left(x,y\right)}^{\mathrm{PFP}}\;={\;s}_{\left(a,x\right)}^{\mathrm{PFP}}\frac{\omega_{\mathrm{xy}}}{\sum_{k\epsilon \tau \left(x\right)}\omega_{\mathrm{xy}}}$$

In the above formula, w_xy_ represents the weight of the link between node pairs, x = staring node then $s_{\left(a,x\right)}^{\mathrm{PFP}}$ = 1, otherwise,${\;s}_{\left(a,x\right)}^{\mathrm{PFP}}$ refers to the set of shortest paths between x and y.

2. Likelihood methods

This section introduces two algorithms based on likelihood methods, including hierarchical structure models and stochastic block models.

- Hierarchical Structure Models (HSM)

The empirical study on the actual network structure shows that, in many cases, the network has a certain hierarchical structure. Moore and Newman [48,49,50] proposed a hierarchical model for predicting missing links, which revealed hidden structural relationships. This method applies to networks with an obvious hierarchy, such as terrorist attack networks and food chain networks. Networks with less hierarchical structures such as co-authorship networks do not perform as well as the simplest CN. From the perspective of link prediction practicability, the computational time complexity of this method is relatively large, and it is unsuitable for large networks.

- Stochastic Block Models (SBM)

Because some networks do not fit in a hierarchical schema [51], another popular and well-known likelihood method is the SBM [52,53], which is based on likelihood estimation. Its basic idea is to group the nodes of the network according to the characteristics of the network’s modularity, and whether two nodes are connected with edges is determined by their group. Stochastic block models can predict the missing edges and judge which are the wrong edges according to the credibility, such as the wrong understanding of protein interaction. RBMs perform better on average than hierarchical models, especially regarding predicting error links. However, it has the same problem of high computational time complexity as the hierarchical model.

3. Probabilistic methods

In complex networks, probabilistic methods are designed to abstract underlying structures from observed structures. It then uses the knowledge to predict the missing link model. The basic idea of it is to establish a model containing a set of adjustable parameters, and then use the optimization strategies to find the optimal parameter values, so that the resulting model can better reproduce the structure and relationship features of the real network, then, the probability of potential link formation is obtained based on the estimated value of the optimal parameter. Representative studies of this method include probabilistic relational, entity-relational, and stochastic relational models. The advantage of the probabilistic model lies in its high prediction accuracy, which uses the structural information of the network and involves the attribute information of nodes. However, the complexity of the calculation and non-universal parameters limit its application scope.

- Probabilistic Relational Models (PRM)

PRM [54] inherits a comprehensive relationship model of the relevant technology and ideas, forming a suitable network forecast model. The model includes classes and relationships. Each entity has some properties, including the types of relationships internal to certain entities and between entities, and each attribute value restrictions in the predefined domain [55]. The model can be divided into the Bayesian network relation model, RBN, RMN, and RDN models by the creation method.

- Entity-relational models (ERM)

The entity-relationship model directly abstracts the data model of entity types and relationships between entities from the real world. The most important and widely used entity-relationship model is the directed acyclic entity-relationship model (DAPER). It is based on the entity-relationship model, which treats the relationship between entities as important. The DAPER [56] model consists of six classes, namely entity, relationship, attribute, arc, local distribution, and constraint class. The entity classes are the entity of the network. Relationship classes describe the relationships between entities, and the attribute classes are attributes of entities or relationships. The arc class is used to describe the relationship between various attributes, and the local distribution class for an attribute class is like the conditional probability in PRM. The constraint class measures the constraints between attribute relationships.

- Stochastic Relational Models (SRM)

SRM is the study of social, physical, and other related phenomena in which interactions between entities can be observed [57]. The key concept of SRM is the random entity process, which is a Gaussian process generated by multiple entities interacting.

(20) $$P(R_{I}|\Theta )\;=\;\int^{\;}\prod_{\left(\mathrm{ij}\in I\right)}P(r_{\mathrm{ij}}|t_{\mathrm{ij}})P(t|\Theta )\mathrm{dt},$$

where R_I_ = r_ij_ ∈ I, and the Θ values are obtained by maximizing the marginal likelihood. Then, a pair of new entity links can predict by marginalizing over the posterior p(t|R_I_, Θ).

#### 3.5.2. Feature Learning Methods

In the computer-generated field, many link prediction methods are based on the features of learning. These methods treat link prediction as a two-classification problem and make x and y as nodes in graph G(V,E), and *l*^(x,y) as labels of node pairs of instances (x,y). In link prediction, each pair of non-connected nodes corresponds to an instance, including class labels and features describing the pair of nodes. Therefore, if there is a link between nodes, the pair of nodes is marked as positive; otherwise, the pair of nodes is marked as negative. The labels for nodes x and y are defined as

(21) $$l^{\left(x,y\right)}\{\begin{array}{c} +1,\;\;\;\mathrm{if}\left(x,y\right)\in E\\ -1,\;\;\mathrm{if}\left(x,y\right)\notin E \end{array}.$$

This paper divides the current feature learning link prediction methods into four categories, namely the supervised learning, semi-supervised learning, unsupervised learning, and reinforcement learning methods.

1. Unsupervised learning

In unsupervised learning, the data used are untagged. That is to say, the output corresponding to the input data is unknown. Unsupervised learning can only read data silently, looking for the models and rules of the data. Therefore, there is no answer for things that you encounter in the process of learning, you have to grope for them and then make classification judgments. The unsupervised method has low complexity and simple calculation. Nowadays, the commonly used unsupervised learning link prediction methods are DeepWalk, large-scale information network embeddings (LINE), GraRep, deep neural graph representations (DNGR), structural deep network embeddings (SDNE), Node2Vec, high-order proximity preserved embedding (HOPE), GraphGAN, and Struct2vec. These methods are detailed below.

- DeepWalk

Bryan et al. [58] introduced the skip-gram model into the study of social networks for the first time and designed the DeepWalk method, which outlines the latest advances in language modeling in learning unsupervised features from word sequences to graphics. The main idea is to construct the random walk path of nodes on the network to simulate the process of text generation, provide a sequence of nodes, and then use word2vec to vectorize each node. DeepWalk generates random walks on demand. Because the skip-gram model is also optimized for each sample, the combination of random walk and skip-gram makes DeepWalk an online algorithm.

- LINE

Tang et al. [59] proposed a novel network embedding method called the LINE, which can be easily extended to the network with millions of vertices and billions of edges. This method uses the breadth-first search strategy to generate context nodes. Compared with layered softmax used in DeepWalk, it uses negative sampling to optimize the skip-gram model. In the actual calculation process, the author introduces a series of pretreatment and optimization methods such as negative sampling to accelerate learning performance. Xu et al. [60] also studied a new LINE method, which also tried to explain and visualize the local update process by using logistic regression, and optimized the local update process by adding prior and intercept to the objective function.

- GraRep

Cao et al. [61] proposed GraRep, which was a graph representation learning based on global structure information. It used the matrix decomposition method to learn node representation. The algorithm considered a special relational matrix and reduced the dimension of the relational matrix through SVD decomposition to obtain the k-step network representation. The graph adjacency matrix is promoted to a different power to use co-occurrence information of nodes of different scales. The singular value is applied to the power of the adjacency matrix to obtain a low-dimensional representation of the node. The disadvantage of GraRep is its poor scalability and low computational efficiency when calculating relational matrices.

- DNGR

Cao et al. [62] proposed a deep neural network model DNGR that encodes each vertex as a low-dimensional vector representation. It uses the L2-Reconstruction method for dimensionality reduction and combines random walk and depth self-encoder. The model consists of three parts, namely random walk, positive point mutual information calculation, and superposition denoising from an encoder. DNGR demonstrated the ability of stacked denoising autoencoders in extracting meaningful information and generating informative representations. DNGR has made great progress in reconstructing network models. The difference between DNGR and SDNE is in the definition of the similarity vector. DNGR takes the common path obtained from random walk of two nodes as the index to measure their similarity, whereas SDNE directly uses the first-order relationship as the input of similarity.

- SDNE

Wang et al. [63] proposed a structured deep network embedding method, namely SDNE, which can effectively capture highly nonlinear network structures and maintain global or local structures. It is supervised and unsupervised and uses two-dimensionality reduction methods, L2-reconstruction and Laplace feature mapping.

- Node2Vec

In 2016, Grover et al. [64] proposed a new link prediction method, the Node2Vec for continuous feature representation of learning network nodes. It combines BFS and DFS to sample the nodes in the graph. They believed that the AUC value of Node2Vec was superior to CN, Jaccard’s coefficient, and AA in link prediction.

- HOPE

Ou et al. [65] proposed a scalable high-order approximate retention-embedding method, HOPE, to solve the problem of asymmetric transitivity in directed graph embedding. This property is essential for capturing the structure of a node when the vertex of a factorization graph is mapped to a vector space. A singular value decomposition is applied to the adjacency matrix. The experimental results show that HOPE outperforms AA, CN, and PPE significantly in link prediction.

- Graph Representation Learning with Generative Adversarial Nets (GraphGAN)

Wang et al. [66] published a paper, proposing GraphGAN. It combines the generative and discriminant model. Under the GraphGAN framework, both the generator and discriminator could benefit from each other: The generator must generate data as similar to the real data as possible to fool the discriminator. The discriminator using the sigmoid function must separate the real data from the generated data as much as possible. Experimental results show that GraphGAN is more accurate than DeepWalk, LINE, Node2Vec, and Struc2Vec. Especially in the field of link prediction, better performance has been obtained.

- Struct2Vec

Ribeiro et al. [67] proposed a new link prediction method for capturing structural features of graphs. Struct2Vec is another biased random walk-based approach. The main difference between Node2Vec and Struc2Vec [68] is that while in Node2Vec, random walks occur on the original network, and in Struc2Vec, they occur on a modified version of the network where close nodes are structurally similar in the original network. This method uses the degree of nodes to evaluate the structural similarity of node pairs and generate the context. Compared to DeepWalk and Node2Vec, the limitation is overcome by structural identity, and Struc2Vec is superior in link prediction tasks.

2. Semi-supervised learning

In semi-supervised learning, few or less data are tagged. Therefore, compared with supervised learning, semi-supervised learning has a lower cost but higher accuracy. This equals training from a small amount of data with answers, and then marking and classifying the rest of the data according to the learning experience. In practice, semi-supervised learning is also frequently used; mostly we lack tagged data. Currently, the commonly used semi-supervised learning link prediction methods are graph convolutional networks (GCN), graph neural network (GNN), generative antagonism network (GAN), long-short-term memory (LSTM), GAE (graph autoencoder), and GAT (graph attention network).

- GCN

Tomas et al. [69] proposed a semi-supervised learning algorithm based on spectral convolution, which can encode the graph structure data and features effectively and has good performance in the field of link prediction. Chen et al. [70] proposed a batched training scheme, FastGCN that defined convolution as a form of integral transformation to solve a common memory bottleneck in GCN. They reconstructed the sampling program of loss and gradient, which is proven by the integration transformation of the embedded function. Numerous experiments have confirmed that FastGCN’s prediction speed is one order of magnitude faster than GNN, and it maintains a high prediction performance. Michael et al. [71] proposed a new link prediction approach, namely relational-GCN (R-GCN). Numerous experiments have proven the prediction performance of this method. Regarding the standard link prediction, a good competition effect is obtained and the factorization model is enriched by R-GCN.

- GNN

Scarselli et al. [72] proposed GNN for complex network link prediction. This is an extension of the neural network approach used to process data in the graph domain. This GNN model can handle graphical data directly, such as acyclic, cyclic, directed, and undirected graphs, and it implements a function τ(G,n) $\in R^{m}$ mapping a graph G and node N entered the m-dimensional Euclidean space. Zhang et al. [73] proposed the capsule graph neural network, adopting the concept of a capsule to solve the problem of the GNN-based graphics embedding algorithm. By extracting node features in the form of capsules, critical information is captured at graph level by a routing mechanism. Singh et al. [74] proposed a novel GNN method that learns both node and edge features as opposed to only the node features under typical GNN. The main advantage of using GNN for link prediction lies in its ability to connect characters that are spatially separated and have an arbitrary orientation.

- GAN

GAN is a deep learning model. By generating network G and discriminating network D, a continuous game is played so that G can learn the distribution of data. If it is used in picture generation, G can generate realistic images from a random number after the training. The discriminator can distinguish between real and fake samples. Compared with most link prediction methods, the training model of GAN adopts an antagonistic training model, including the generative and discriminant models. Lei et al. [75] proposed a nonlinear model GCN+GAN to solve link prediction tasks in weighted dynamic networks. It uses the advantage of GCN, LSTM, and GAN. We can improve link prediction performance by using dynamic topology and the time evolution model. Specifically, the authors used GCN to mine topological features, LSTM to describe the time evolution features of the network, and GAN to generate the next weighted network snapshot, effectively solving the problem of sparsity.

- LSTM

The LSTM network is both short-term and long-term memory proposed in 1997 by Hochreiter and Schmidhuber [76]. LSTM is a special type of RNN that can learn long-term dependencies, performing well in affective analysis and text prediction tasks. It is often used for link prediction in complex networks. Chen et al. [77] proposed GC-LSTM, which includes two technologies, GCN and LSTM. It was the first time to combine GCN with LSTM for complex dynamic network link prediction. In this new model, GCN can learn the topology of the network, whereas LSTM can learn the time features. It can also predict deleted and added links.

- GAE

GAE is composed of graph autoencoders (AE) and variational autoencoders (VAE) [78]. Thomas proposed [78] the variational graph auto-encoder and used a GCN encoder and an inner product decoder. The experimental results show that this model has some advantages in citation network link prediction.

- GAT

GAT uses the attention mechanism to weight and sum the features of neighboring nodes. The weight of the features of neighboring nodes depends on the features of the nodes, independent of the graph. Gu et al. [79] proposed DeepLinker methods, which extend the GAT architecture to be applied to predict the missing links over various networks. With the attention mechanism, DeepLinker can extract meaningful vertex representation and achieve state-of-the-art link prediction accuracy.

3. Supervised learning

Supervised learning is the process of using labeled or ground truth datato the machine to learn. Supervised methods have features and labels. In real life, link prediction uses supervised learning more, including support vector machine (SVM), K-nearest neighbor (KNN), logistic regression (LR), ensemble learning (EL), random forrest (RF), multilayer perceptron (MP), naïve Bayes (NB), and matrix factorization (MF). Hasan et al. [80] used the supervised method to solve the link prediction problem surprisingly effectively. Numerous experimental results and data confirmed that the commonly used methods based on supervised learning (SVM, decision tree, KNN, multilayer perceptron) have good performance in the field of link prediction, but SVM wins with a slight advantage.

- SVM

SVM used a kernel function to transform a nonlinear problem into a linear one. Its core idea is to find a decision boundary that maximizes the sample interval so that the two types of samples fall on both sides of the surface as far as possible. Its advantage is that SVM has strong robustness despite overfitting problems, especially in high-dimensional spaces. The disadvantage is that choosing the right kernel is complicated and does not apply to large data sets. Jalili et al. [81] designed a new multiplexing link prediction technology on Twitter and Foursquare, namely the link prediction method based on meta-path, adopting three commonly classical classifiers (SVM, naïve Bayes, and KNN) to predict a link. Laishram et al. [82] extended the current link prediction method for weighted and directed networks using SVM to predict links between nodes.

- KNN

Using a distance function (Euclidean distance, Manhattan distance, or Hamming distance), KNN searches for the most similar training sample to predict the observed value of the new sample. Its advantages are simple calculation, easy to understand, easy to implement, high precision, and insensitive to outliers. Aouay et al. [83] combined different input features and used a supervised learning method to conduct link prediction. We improved the performance and accuracy of link prediction using the selection attribute method. Experiments show that the comprehensive performance of random forest, KNN, and principal component analysis is the best.

- Logistic Regression (LR)

The LR method is a generalized linear model, judging whether a model is linear or not. The interface of LR is linear. Its advantages are low complexity and easy to implement, but its disadvantages are easy to produce underfitting and low classification accuracy. Zhou et al. [84] used the dynamic LR method for dynamic network link prediction. Chiang et al. [85] used a LR predictor of link prediction in signed networks, which realized accurate sign prediction and were effective for reducing false-positive rates.

- Ensemble Learning (EL)

EL is a commonly used method of supervised learning. It is a meta-algorithm combining several machine learning techniques into a prediction model to reduce variance or improve prediction. Currently, ensemble learning can be used for classification problem integration, regression problem integration, feature selection integration, and anomaly point detection integration, which can be seen in all machine learning fields. Pachaury et al. [86] used random forest classifiers in integrated learning models to predict missing links in social networks. Guns et al. [87] proposed ensemble learning combined with the random forest (RF) method, constructing three periods of tuberculosis between 1997 and 2011 in the field of the weighted network of cooperation. Using the ensemble learning build classifier, the recommendation accuracy is better than that of a single index.

- RF

RF is a flexible and easy-using supervised learning algorithm that can perform regression and classification tasks. It is also a means of data dimensionality reduction, which is used to deal with missing values, outliers, and other critical steps in data exploration. It uses traversal algorithms to obtain the optimal weight combination of classification results, selects the prediction of top accuracy as the recommendation result and achieves superior results in the field of link prediction. Scellato et al. [88] used geographic location, social, and global features, and naïve Bayes and RF to predict social networks based on geographic locations and achieved high prediction accuracy.

- Multilayer Perceptron (MLP)

MLP is a generalization of perceptron. It is characterized by multiple layers of neurons, so it is also a deep neural network. A critical feature of the multilayer perceptron is the multilayers. The first layer is the input layer, the last layer is the output layer, and the middle layer is the hidden layer. MLP does not specify the number of hidden layers, so you can choose the appropriate number of hidden layers according to your needs. There is no limit on the number of neurons in the output layer. Kastrin et al. [89] used the multilayer perceptron to predict time-series. In the backpropagation algorithm, the weight is initialized with a small random value, and the appropriate weight initialization will produce a good solution to the weight approach; thus, reducing the training time. Experimental data show that proper weight initialization can obtain better prediction performance.

- Naïve Bayes (NB)

NB is a simple probabilistic classifier that must solve the probability of each category under the condition of the occurrence of a given item to be classified. The greater the value is, the category belongs to this category. It has the advantage of being easy to implement and useful for large data sets. Supporting incremental operations means training new samples in real-time. Its disadvantage is that it assumes sample attribute independence, so it is less effective if the sample attribute is associated. Hasan et al. [4] discussed several commonly used link prediction approaches and classified them. Second, it uses the Bayesian method for modeling. Finally, the reduced rank similarity matrix is used to calculate the similarity between nodes. Jorge et al. [90] used the overlapped topological information to build a Bayesian model for link prediction on complex networks.

- Matrix Factorization (MF)

Links between nodes can also be represented as adjacency matrices, where each row and column represent a different node and Boolean variable to show whether there are links between node pairs. The link prediction is a matrix completion problem, and the matrix decomposition method is extended to solve the link prediction problem. Kunegis et al. [91] proposed a method of algebraic spectrum transformation based on a network adjacency matrix to predict the existence of links in the network and link weights. This method has much fewer parameters to learn than other methods, but the computational complexity is higher. Aditya et al. [92] proposed a supervised MF method to solve the link prediction problem, which learns latent features from the topological structure of a graph and combines it with optional explicit features for nodes or edges. The experimental results showed that this method makes better predictions than popular unsupervised methods. The advantage of MF considers global node proximity, but it has considerable time and space consumption.

4. Reinforcement Learning Methods

Reinforcement learning is intermediate between supervised and unsupervised learning. It records the results of different actions and tries to make decisions using the machine’s history and experience. It has a partial feedback for each prediction or behavior. It adopts the method of learning while acquiring samples. After obtaining samples, it updates its model and uses the current model to guide the next action. After the next action obtains results, it updates the model and iterates or repeats until the model converges. Reinforcement learning methods include graph convolutional policy network (GCPN) and graph Transformation Policy Network (GTPN).

- GCPN

You et al. [93] proposed GCPN, which models graph generation as a Markov decision process and uses the generated model as a reinforcement learning agent that runs in the graph generation environment. GCPN uses similar agent action as link prediction problems, uses domain-specific and confrontation rewards, and uses GCN to learn node representations, thereby implementing end-to-end training through a strategy gradient method. Comparative experiments found that GCPN is effective in graph generation problems and has superior performance in link prediction.

- GTPN

Do et al. [94] proposed GTPN, which used reinforcement learning to predict chemical reaction products. The agent acts by selecting pairs of nodes in the molecular graph and predicting their new bonding types, with immediate and final rewards based on whether the predictions are correct. GTPN uses GCN to learn node representation and RNN memory prediction sequences.

### 3.6. Selection Layer

The selection layer is located in the sixth layer of the seven-layer model. It selects one or more link prediction methods from the upper layer to complete the link prediction task.

(1) Single method

The single method is to choose a method from the sixth layer for link prediction. Currently, most link prediction articles adopted the single method. For instance, Zhang et al. [95] published an article named link prediction based on graph neural networks, which only used GNN for link prediction.

(2) Combination methods

Combination methods are selected from the commonly used link prediction methods in the sixth layer to perform link prediction on complex networks simultaneously. The advantage of this combination method is to exploit the advantages and avoid the disadvantages. Lei et al. [75] combined GCN, GAN, and LSTM for link prediction of weighted dynamic networks exploiting all advantages of the three methods. It combines dynamics, topology information, and evolutionary models to improve the performance of weighted dynamic network link prediction.

### 3.7. Output Layer

The output layer is the last layer of the seven-layer model, which outputs the results obtained from the upper processing layer using the link prediction algorithm. This layer outputs a group of low-dimensional vectors to represent the graph. The output types of link prediction are divided into three categories, namely edge, subgraph, and whole-graph.

The first method outputs edge, and the prediction results show whether there is a link between the two nodes. The results apply link and entity-relationship prediction in the knowledge graph. In the application of link prediction, we will also output a feature for each edge and use it as the feature of edge to perform some classification tasks in subsequent work. The second method outputs subgraphs, including substructures. The third is the whole-graph, that is, an entire graph to output. The whole-graph usually applies to small graphs, such as proteins and molecules.

## 4. Comparison of Input Features of Link Prediction Methods and Complexity

This part classifies and summarizes the input metadata features used in the link prediction methods discussed above (Table 1). We also analyze the complexity of these methods in detail (Table 2).

## 5. Evaluating Metrics

The performance evaluation of link prediction in complex networks is critical in the research of link prediction models. Common evaluation indicators include AUC and precision, which measure the performance of link prediction algorithms at different levels. Among them, AUC is the most used evaluation index and can measure the performance of the prediction algorithm on the whole. The precision only focuses on the prediction accuracy of unknown edges with the highest prediction scores.

1. AUC

As one of the methods to measure the overall predictive structure model, AUC has the advantages of global and accuracy. It can be understood as the probability that the score value of the edge in the test set is higher than the score value of the randomly selected non-edge, that is, each time the test set is compared with the randomly selected non-existent edge. If the value of the edge in the test set is greater than the score value of the non-existent edge, add 1 point. If the two scores are equal, we add 0.5. It compares independently n times. If the score of the edge in the test set is greater than the score of the non-existent edge by n′ times, and there are n″ times when the two points are equal, the AUC value is defined as follows:

(22) $$\mathrm{AUC}\;=\;\frac{n^{\prime }+0.5\;n^{\prime \prime }}{n}.$$

Obviously, if all the scores are randomly generated, then AUC = 0.5, so the degree of AUC greater than 0.5 measures how accurate the algorithm is than the randomly selected method.

2. Precision

Precision represents the accuracy index of link prediction. It refers to selecting the top L positions after the unknown edge set is sorted in descending order of score. If m edges belong to the test set, precision can be expressed as:

(23) $$\mathrm{Precision}\;=\;\frac{m}{L}.$$

## 6. A Summary of Open-Source Implementations

This part summarizes the source code of the above common link prediction methods to avoid the duplication of research and improve the efficiency of research (Table 3).

## 7. Experimental Comparison and Relative Merits for Each Link Prediction

In this part, we conduct an experimental comparison of the link prediction algorithms involved in this article, and analyze their relative merits (Table 4). At the same time, we present common datasets, so that the reader can reproduce the experiments (Table 5).

## 8. Future Directions

In the last two decades, experts from computer sciences, physics, biology, and other disciplines in the field of link prediction research have produced many different research results according to their discipline characteristics. These results promote the development of link prediction in theory and solve many practical problems. Some challenging issues raised in the past have been advanced to varying degrees. There will be more extensive attempts in the future, of which we list seven possible research directions.

1. Link prediction for complex type networks
  Existing research is imperfect, opening the opportunity to explore how to make link predictions in complex network structures, such as multiple layer networks, interdependent networks, and hypernetworks.
2. Personal privacy protection
  User privacy protection is an unavoidable problem in practical applications. How to obtain accurate prediction effects without compromising user privacy is also a problem worthy of study.
3. Interpretability
  Link prediction has many practical applications, making it critical to explain the prediction results. In medicine, such interpretability is essential in translating computer experiments into clinical applications.
4. Combination
  As mentioned above, many existing methods can work together. How to fully exploit the advantages of each method and combine them should be solved.
5. Scalability and parallelization
  In the era of big data, large social networks typically have millions of nodes and edges. Therefore, designing an extensible model with linear time complexity becomes critical. Furthermore, because the nodes and edges of a graph are interconnected, it is often necessary to model it in its entirety, highlighting the need for parallel computation.
6. Interdisciplinary
  Link prediction has attracted the attention of experts in various fields. Interdisciplinary crossing brings both opportunities and challenges. Domain knowledge is used to solve specific problems, but cross-integration domain knowledge could make the model design more difficult.
7. New evaluation methods
  Exploring new evaluation criteria is also an important issue that needs to be solved in future work [96]. Using the diffusion process in dynamic networks to evaluate link prediction methods is a promising research direction in the future [97].

## 9. Summary

Link prediction in complex networks has received much attention from experts in various fields. This paper defined link prediction and proposed a seven-layer model to classify link prediction methods. These methods cover many classic and latest link prediction techniques, namely the similarity-based, probabilistic, likelihood, unsupervised learning, semi-supervised learning, supervised learning, and reinforcement learning methods. Furthermore, the evaluating metrics, input features, complexity, experimental comparisons, relative merits, common dataset, and open-source implementation are analyzed in detail. Through feature analysis and experimental comparison, we found that the link prediction method based on graph structure features is better than other link prediction methods. Finally, future directions and challenges are addressed.

## Figures and Tables

**Table 1:** The input features of common link prediction methods are summarized.

Algorithms	Time	Topology	Weight	Attributive	Label	Directional	Symbolic	Auxiliary Information
Katz [31]		√						
SR [32]		√		√				
RW [22]		√		√		√		
RWR [33]		√		√				√
CN [34]		√		√				√
JC [36]		√		√				
SI [38]		√						
PA [39]	√	√						
AA [40]		√						√
RA [41]		√						
HPI [42]		√						
HDI [43]		√						
LP [44]		√						
LRW [45]		√						
SRW [45]		√						
FL [46]		√						
PFP [47]		√						
HSM [48]		√						√
SBM [51]		√						√
PRM [55]		√		√				
ERM [56]		√						
SRM [57]	√	√		√				
DeepWalk [58]		√			√			√
LINE [59]		√	√			√		
GraRep [61]		√	√					√
DNGR [62]		√	√					
SDNE [63]		√						
Node2Vec [64]		√			√			√
HOPE [65]		√				√		
GraphGAN [66]		√						
Struct2vec [67]	√	√	√	√	√			√
GCN [69]		√		√	√			√
GNN [72]		√		√	√			√
GAN [75]	√	√	√					
LSTM [76]	√	√		√				√
GAT [79]		√		√				
GAE [78]		√		√		√		
SVM [81]		√	√	√		√		
KNN [83]		√		√				√
LR [84]	√	√	√		√		√	√
EL [86]		√	√	√				√
RF [88]		√		√				√
MLP [89]	√	√	√	√				
NB [90]		√		√				√
MF [91]		√		√		√		
GCPN [93]		√		√		√		
GTPN [94]	√	√		√				

**Table 2:** Complexity analysis for link prediction methods.

Category	Algorithm	Complexity	Remarks
**Similarity-based methods**	Katz [31]	O(N^3^)	N represents the number of nodes in the network.
	SimRank [32]	O(N^4^)	
	Random Walk [22]	O<cN^2^k>	C is the network aggregation coefficient. K stands for average degree.
	Random Walk with Restart [33]	O(N^3^)	
	Common Neighbors [34]	O(N^2^)	
	Jaccard Index [36]	O(2N^2^)	
	Salton Index [38]	O(N^2^)	
	PA [39]	O(2N)	
	AA [40]	O(2N^2^)	
	RA [41]	O(2N^2^)	
	Hub Depressed index [42]	O(N^2^)	
	Hub Promoted index [43]	O(N^2^)	
	Local Path index [44]	O(N<K>^3^)	K stands for average degree.
	LRW [45]	O(N<K>^n^)	n represents the number of random walk steps.
	SRW [45]	O(N<K>^n^)	
**Unsupervised learning Methods**	DeepWalk [58]	O(|V|d)	|V| represents the number of nodes in the graph. d represents the average shortest distance.
	LINE [59]	O(|E|d)	|E| represents the number of edges in the graph.
	GraRep [61]	O($\left|V\right|\left|E\right|+d{\left|V\right|}^{2}$)	
	DNGR [62]	O(${\left|V\right|}^{2}$)	
	SDNE [63]	O(|V||E|)	
	Node2Vec [64]	O(|V|d)	
	HOPE [65]	O($\left|E\right|d^{2}$)	
	GraphGAN [66]	O(|V|log|V|)	
	Struct2vec [67]	O(${\left|V\right|}^{3}$)	
**Semi-supervised learning Methods**	GCN [69]	O($\left|E\right|d^{2}$)	
	GNN [72]	O($\left|E\right|d^{2}$)	
	GAN [75]	O(|V|log|V|)	
	LSTM [76]	O(nm+n^2^+n)	n is hidden_size, m is input_size.
**Supervised learning Methods**	SVM [81]	O(n^2^)	n is the number of samples.
	KNN [83]	O(n*k*d)	d is data dimension, k is the number of neighbors.
	Logistic Regression [84]	O(n*d)	
	Ensemble learning [86]	O(n)	
	Random Forrest [88]	O(n*log(n)*d*k)	
	Naïve Bayes [90]	O(n*d)	

**Table 3:** A summary of open-source implementations.

Model	Framework	Github Link
Random walks [22]	python	https://github.com/tblee/Link_prediction
RWR [33]	python	https://github.com/TuftsBCB/Walker
Similarity based methods [34,35,36,37,38,39,40,41,42,43,44,45]	python	https://github.com/CodeZWT/Link-Prediction
Hierarchical structure method [48]	matlab	https://github.com/joana92/Link-prediction-in-network
Stochastic Block Models [51]	python	https://github.com/dongwookim-ml/MMSB
DeepWalk [58]	python	https://github.com/phanein/deepwalk
LINE [59]	python	https://github.com/tangjianpku/LINE
GraRep [61]	python	https://github.com/benedekrozemberczki/GraRep
DNGR [62]	matlab	http://github.com/ShelsonCao/DNGR
SDNE [63]	python	http://github.com/suanrong/SDNE
Node2vec [64]	python	https://github.com/adocherty/node2vec_linkprediction
HOPE [65]	matlab	https://github.com/ZW-ZHANG/HOPE
GraphGAN [66]	python	https://github.com/hwwang55/GraphGAN
Struct2Vec [67]	python	https://github.com/leoribeiro/struc2vec
Link prediction based on unsupervised method (DeepWalk, LINE, Node2vec, SDNE, Struc2vec)	python	https://github.com/shenweichen/GraphEmbedding
GCN [69]	python	https://github.com/tkipf/gcn
GNN [72]	python	https://github.com/weihua916/powerful-gnns
GAN [75]	python	https://github.com/jhayes14/GAN
LSTM [76]	matlab	https://github.com/huashiyiqike/LSTM-MATLAB
Link prediction based on supervised method	python	https://github.com/alpecli/predlig
GAE [78]	python	https://github.com/tkipf/gae
GCPN [93]	python	https://github.com/bowenliu16/rl_graph_generation

**Table 4:** Experimental comparison and advantages of link prediction method.

Classification	Methods	AUC	Precision	Dataset	Relative Merits
**Similarity-based methods**	Katz	0.956	0.719	USAir	Katz sums over the sets of paths.
	RWR	0.978	0.650	PPI	RWR provides a good relevance score between two nodes in a weighted graph.
	CN	0.937	0.814	USAir	CN is simple and intuitive.
	JC	0.933		NS	JC normalizes the size of CN.
	SI	0.911		NS	SI is the metric which is known as cosine similarity in the literature.
	PA	0.886	0.764	USAir	PA has the lowest complexity compared with other algorithms and requires the least information.
	RA	0.955	0.828	USAir	RA is more superior when the average degree is high.
	AA	0.932	0.699	NS	AA refines the simple counting of CN.
	HPI	0.911		NS	HPI value is determined by the lower degree of nodes.
	HDI	0.933		NS	HDI value is determined by the higher degrees of nodes.
	LP	0.939	0.734	PB	LP has obvious advantage in computing speed for the large and sparse network.
	LRW	0.989	0.863	NS	LRW was suitable for large and sparse networks.
	SRW	0.992	0.739	NS	SRW optimizes prediction accuracy at an earlier time and prevent sensitive dependence of LRW to the nodes further away.
	FL	0.875		Epinions	FL can provide more accurate and faster link prediction.
	PFP	0.917		Ca-condmat	PFP is highly scalable and achieves major improvements.
**Likelihood methods**	HSM	0.856		Food	HSM is suitable for networks with obvious hierarchical structure.
	SBM	0.902		Ca-condmat	SBM is suitable for predicting error edges.
**Probabilistic method**	PRM	0.874		WebKB	PRM can be significantly improved by modeling relational dependencies.
	ERM	0.889		DBLP	ERM are capable of performing better than the other models when the relational structure in uncertain.
	SRM	0.942		Movie	SRM can reduce the overall computational complexity.
**Unsupervised learning methods**	DeepWalk	0.809	0.839	Blogcatalog	DeepWalk can generate random walks on demand, it is efficient and parallelized.
	LINE	0.837	0.814	DBLP	LINE is suitable for arbitrary types of information networks and improves both the effectiveness and the efficiency of the inference.
	GraRep	0.814		Blogcactalog	GraRep can capture global structural information associated with the graph and extend it to support weighted graphs.
	DNGR	0.804		Wikipedia	DNGR can capture nonlinear information conveyed by the graph and learn better low-dimensional vertex representations of graph.
	SDNE	0.836		Arxiv	SDNE can capture the highly nonlinear network structure and is robust to sparse networks.
	Node2Vec	0.968	0.854	Facebook	Node2vec is flexible, controllable, scalable, and robust.
	HOPE	0.881	0.812	Twitter	HOPE is scalable to preserve high-order proximities of large-scale graphs and capable of capturing the asymmetric transitivity.
	GraphGAN	0.859	0.853	Arxiv	GraphGAN achieves substantial gains in link prediction and satisfy desirable properties of normalization.
	Struct2Vec	0.853	0.810	Air-traffic network	Struct2Vec can capture stronger notions of structural identity.
**Semi-supervised learning methods**	GCN	0.941		Citeseer	GCN can effectively encode graph structure data and features and achieve high prediction speed and performance.
	GNN	0.890	0.891	Neural netwok	GNN can capture more local structure information, provide much richer representation and calculates faster.
	GAN	0.932	0.920	UCSB	GAN only uses backpropagation, without the need for a complicated Markov chain, and it can generate samples that are clearer and more realistic than other models.
	LSTM	0.982	0.810	Hypertext	LSTM can fit sequence data and solve the problem of gradient disappearance.
	GAE	0.925	0.902	Core	GAE can address link prediction in directed graphs.
	GAT	0.880	0.790	Core	GAT can not only make predictions on links but also learn meaningful node representations.
**Supervised learning methods**	SVM	0.982	0.991	Facebook	SVM is extremely robust, especially in high-dimensional spaces.
	KNN	0.803	0.920	Flickr	The theory of KNN is simple and easy to implement, new data can be added directly without retraining.
	LR	0.901		Epinions	LR is not computationally expensive and easy to understand and implement.
	EL	0.994	0.989	Flickr	EL combines various classifiers to learn from each other and has better prediction performance.
	RF	0.987	0.989	Facebook	RF can achieve high accuracy, without worrying about overfitting, each time only a few randomly selected features are used to train the tree.
	MLP	0.862		Mesh	MLP can learn nonlinear models and can perform real-time learning.
	NB	0.808	0.04	Flickr	NB is easy to implement and very useful for large data sets.
	MF	0.793		PowerGrid	MF has much fewer parameters to learn and capture global structure.
**Reinforcement learning methods**	GCPN	0.855	0.741	ZINC250K molecule dataset	GCPN is effective in a variety of graph generation problems, especially in dealing with link prediction problems, and has better performance.
	GTPN	0.906	0.832	USPTO	GTPN improves the top-1 accuracy over the current state-of-the-art method by about 3% on the large USPTO dataset.

**Table 5:** Popular open datasets used in link prediction.

Data Source	Description	Nodes	Edges	Sites of Datasets
C.elegans	elegans worm	306	2345	https://toreopsahl.com/datasets/#celegans
PPI	Protein interaction	2617	11,855	http://snap.stanford.edu/graphsage/ppi.zip
USAir	transport	1574	28,236	https://toreopsahl.com/datasets/#usairports
PowerGrid	American power network	4941	6595	http://www-personal.umich.edu/~mejn/netdata/
NS	Scientists cooperation network	1461	2742	http://www-personal.umich.edu/~mejn/netdata/
Movie	Star-director-film-writer network	348	2,332,341	https://www.aminer.cn/data-sna#Twitter-Dynamic-Net
PB	Political blog network	1224	19,090	http://www-personal.umich.edu/~mejn/netdata/
Food	FoodWebs	128	2137	http://vlado.fmf.uni-lj.si/pub/networks/data/bio/foodweb/foodweb.htm
Epinions	Who-trusts-whom network of epinions	75,879	508,837	http://snap.stanford.edu/data/soc-Epinions1.html
Amazon	Amazon product network	334,863	925,872	http://snap.stanford.edu/data/com-Amazon.html
Live Journal	Live Journal online social network	3,997,962	34,681,189	http://snap.stanford.edu/data/com-LiveJournal.html
Blogcatalog	Social relationship nework	88,800	2,100,000	http://networkrepository.com/soc-BlogCatalog.php
Vote	Wikipedia who-votes on whom network	7115	103,689	http://snap.stanford.edu/data/wiki-Vote.html
DBLP	DBLP co-authorship network	317,080	1,049,866	http://snap.stanford.edu/data/com-DBLP.html
Arxiv	Paper citation network of Arxiv	34,546	421,578	http://snap.stanford.edu/data/cit-HepPh.html
Air-traffic network	American air-traffic network	1190	13,599	https://transtats.bts.gov/
Patents citation	Us Patent citation network	3,774,768	16,518,948	http://snap.stanford.edu/data/cit-Patents.html
Facebook	Social circles from facebook	4039	88,234	http://snap.stanford.edu/data/egonets-Facebook.html
Twitter	Lists from Twitter	81,306	1,768,149	http://snap.stanford.edu/data/egonets-Twitter.html
Wikipedia	Vote network	7115	103,689	http://snap.stanford.edu/data/wiki-Vote.html
Slashdot	Signed network	82,140	549,202	http://snap.stanford.edu/data/soc-sign-Slashdot090221.html
email -Enron	Email network	36,692	183,831	http://snap.stanford.edu/data/email-Enron.html
Cit-HepPh	Arxiv high energy physics paper citation network	34,546	421,578	http://snap.stanford.edu/data/cit-HepPh.html
Citeseer	Citation network	3327	4732	http://www.cs.umd.edu/~sen/lbc-proj/data/citeseer.tgz
Cit-HepTh	Arxiv high energy Physics paper citation network	27,770	352,807	http://snap.stanford.edu/data/cit-HepTh.html
UCSB	Wireless mesh net link quality	38	2000	http://crawdad.org/ucsb/meshnet/20070201
Hypertext	Face-to-face contact	113	20,800	http://www.sociopatterns.org/datasets/hypertext-2009-dynamic-contact-network/
Flickr	Social network	2,302,925	33,140,017	http://networkrepository.com/soc-flickr-und.php
Cit-Patents	Citation network among us patents	3,774,768	16,518,948	http://snap.stanford.edu/data/cit-Patents.html
Core	Cite network	2708	5429	https://linqs.soe.ucsc.edu/data
Ca-AstroPh	Collaboration network	18,772	198,110	http://snap.stanford.edu/data/ca-AstroPh.html
Ca-CondMat	Collaboration network	23,133	93,497	http://snap.stanford.edu/data/ca-CondMat.html
Web-Google	Web graph from Google	875,713	5,105,039	http://snap.stanford.edu/data/web-Google.html
Web-Stanford	Web graph of Stanford edu	281,903	2,312,497	http://snap.stanford.edu/data/web-Stanford.html
